# How does unemployment affect self-assessed health? A systematic review focusing on subgroup effects

**DOI:** 10.1186/1471-2458-14-1310

**Published:** 2014-12-22

**Authors:** Fredrik Norström, Pekka Virtanen, Anne Hammarström, Per E Gustafsson, Urban Janlert

**Affiliations:** Department of Public Health and Clinical Medicine, Epidemiology and Global Health, Umeå University, SE-901 85 Umeå, Sweden; School of Health Sciences and Institute of Advanced Social Research, University of Tampere, Tampere, Finland; Department of Public Health and Clinical Medicine, Family Medicine, Umeå University, Umeå, Sweden

**Keywords:** Age, Education, Employment, Gender, Health, Marital status, Review, Subgroups, Unemployed

## Abstract

**Background:**

Almost all studies on the effect on health from unemployment have concluded that unemployment is bad for your health. However, only a few review articles have dealt with this relation in recent years, and none of them have focused on the analysis of subgroups such as age, gender, and marital status. The objective of our article is to review how unemployment relates to self-assessed health with a focus on its effect on subgroups.

**Methods:**

A search was performed in Web of Science to find articles that measured the effect on health from unemployment. The selection of articles was limited to those written in English, consisting of original data, and published in 2003 or later. Our definition of health was restricted to self-assessed health. Mortality- and morbidity-related measurements were therefore not included in our analysis. For the 41 articles included, information about health measurements, employment status definitions, other factors included in the statistical analysis, study design (including study population), and statistical method were collected with the aim of analysing the results on both the population and factor level.

**Results:**

Most of the studies in our review showed a negative effect on health from unemployment on a population basis. Results at the factor levels were most common for gender (25 articles), age (11 articles), geographic location (8 articles), and education level (5 articles). The analysis showed that there was a health effect for gender, age, education level, household income, and geographic location. However, this effect differed between studies and no clear pattern on who benefits or suffers more among these groups could be determined. The result instead seemed to depend on the study context. The only clear patterns of association found were for socioeconomic status (manual workers suffer more), reason for unemployment (being unemployed due to health reasons is worse), and social network (a strong network is beneficial).

**Conclusions:**

Unemployment affects groups of individuals differently. We believe that a greater effort should be spent on specific groups of individuals, such as men or women, instead of the population as a whole when analysing the effect of unemployment on health.

**Electronic supplementary material:**

The online version of this article (doi:10.1186/1471-2458-14-1310) contains supplementary material, which is available to authorized users.

## Background

Many studies have investigated the consequences on self-assessed health from unemployment [1–41], including review articles [42–44] and meta-analyses [45–47]. In the meta-analyses, unemployment has been shown to have a negative effect on health with an effect size ranging from 0.36 to 0.57 (considered as small-sized to medium-sized effects). Most of the other literature in the field is in agreement in suggesting that unemployment is bad for your self-assessed health.

Meta-analyses and review papers on health and unemployment have primarily focused on measuring population-based effects on health from unemployment. For example, even though the meta-analyses by McKee-Ryan et al. [45] and Paul and Moser [46] present results for so-called moderators, the main effort of their work was to derive an effect size for the population as a whole. It is common when studying unemployment and health that moderators are only included as part of the statistical model to improve estimates. If the effect from unemployment on health differs for different levels of the moderator this might lead to misleading results on the population level even if the estimates are unbiased. This is due to the fact that the estimates depend on the interactions of factors such as reasons for unemployment, type of work contract, and different personal characteristics (age, gender, marital status, etc.). Estimates might, therefore, not reflect the situation for most of the groups in the population and it might be advisable to only present stratified estimates.

Despite many previous meta-analyses and reviews in the field, there is still a need for summarizing what has been done in the past, especially because the few reviews published in recent years have not covered the same area as us [42, 43]. More importantly, no review has been undertaken with the same emphasis as ours on characteristics such as age, gender, and education level for which unemployment might affect individuals to a different extent. We wanted to investigate how these and other factors have been dealt with in the past in this field.

Thus, the aim of our article is to review how unemployment relates to self-assessed health, mainly with a focus on its effects on subgroups, such as age, gender, and marital status.

## Methods

For our review, we have chosen articles consisting of original data that sought to measure the effect of unemployment on health. Our definition of health was restricted to self-assessed health and we have not included mortality- and morbidity-related measurements. Our requirement was that the unemployed should be actively searching for a job and not be disabled or retired (a similar definition of unemployment is used in most articles that study unemployment). To be part of our review, the article must have focused on the comparison of unemployed and employed individuals within a population that was not limited to a specific occupation or disease cohort. Thus, the main focus of our included articles should be unemployment in relation to health. In addition to the specifications above, we limited our selection of articles to those written in English and published from 2003 to April 2014. Our review was performed based on the checklist from the Prisma Statement [48], see Additional file 1.

**Figure 1:**
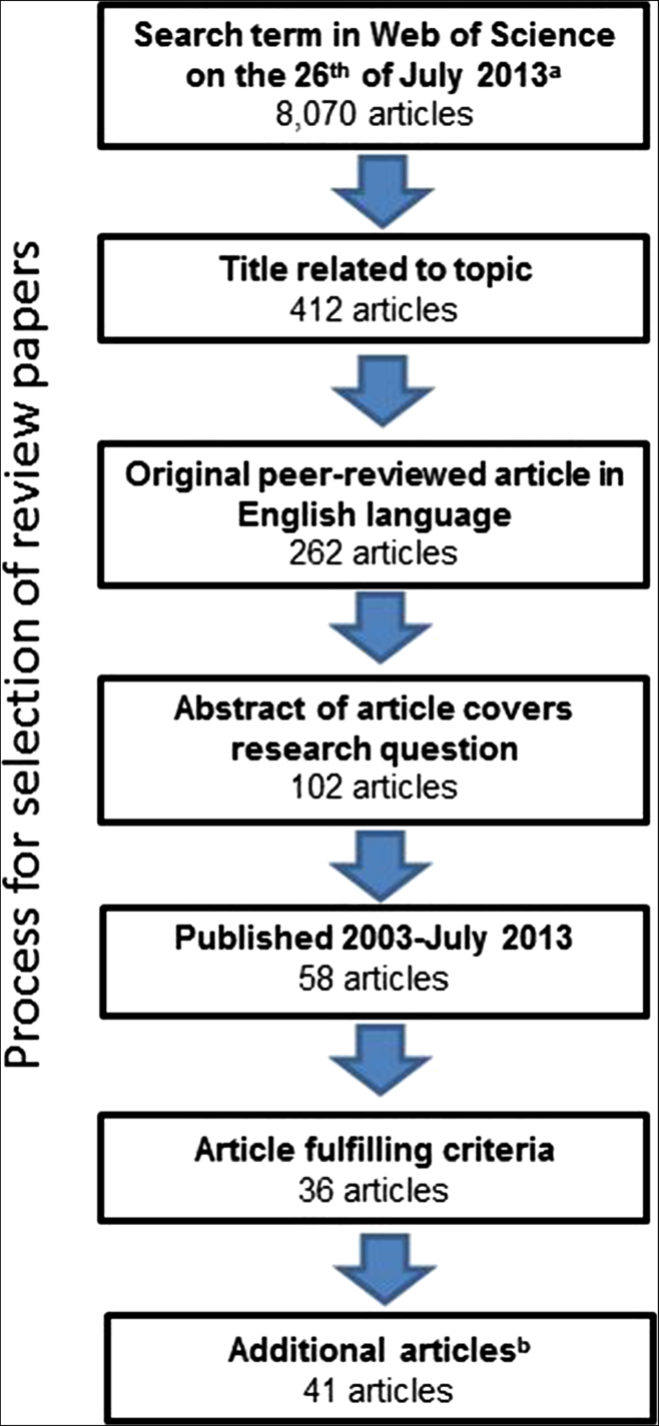
**PRISMA flow diagram.** The flowchart is showing the process in which articles were selected for the review. ^a^see Additional file 2 for search term, ^b^three articles published after initial scanning, one article fulfilling extended search term, and one article based on additional search in PubMed were added.

Full details of the process leading to the final selection of the 41 articles [1–41] are available in Additional file 2. In short, we searched for articles in the literature database Web Of Science (Thomson Reuters) with the following search term: (“well-being” OR “health”) AND (“labor” or “labour” or “employment” or “job” or “unemployment” or “work” or “unemployed”). Articles were only selected if the search term was in the title of the article. We narrowed the list down from 8,070 articles to 36 articles in four subsequent steps based on i) relevance of the title of the article, ii) language (English) and document type (research article), iii) the content of the abstract, and iv) the content of the full article (Figure 1). After this procedure, we scanned Web of Science by adding the term “quality of life” to the health definition (left side) and contract to the employment terms (right side) of the search term. We thereafter checked weekly emails from Web of Science based on the extended search term for new articles until we were late in the writing process. We also performed an additional search in the literature database PubMed (National Center for Biotechnology Information, Bethesda MD, USA) on the MeSH terms “employ* and health”, which only resulted in one additional article compared to the Web of Science search. Thus, the final selection of 41 articles consisted of 36 articles from the extensive search, one from the complementary search, three recent publications, and one publication only available in PubMed. The screening process for articles was completed by one of the authors (FN). We did not perform any quality related criterion for these articles to further restrict the number of articles selected as we wanted to avoid publication bias. Where we consider it motivated, we have commented about weaknesses in studies to highlight when conclusions from the review could have potentially been affected.

From the selected articles, information about: health measurements, employment status definitions, other factors included in statistical analysis, study design (including study population), and statistical methods were collected. For each factor, such as age and gender, we also specified if it was part of the statistical model and if stratified results were presented. Articles that included an interaction term between employment status and a moderator were also handled as stratified results. From every article we extracted odds ratios, as well as other ratio measurements, and absolute difference measurements for unadjusted and adjusted estimates from the articles. When we present adjusted estimates in our review it is for the model with most “controlling” variables.

We first evaluate the results for the articles that have presented non-stratified results on the effect of unemployment on health. Thereafter, stratified results are analysed to determine if there is difference between factor levels. Most articles in our review did not focus on comparing different factor levels with statistical tests. Therefore our interpretations are based on comparing point estimates in the respective subgroups.

## Results

Characteristics of each of the 41 articles in our review are presented in Table 1 and summarized in Table 2. Most studies in our review were from Europe (80%) with other continents having no more than three studies. A majority of the studies used a cross-sectional design (59%), and the rest were longitudinal studies (41%). A wide variety of health measures, most of them validated, were used in our selection of articles. The most common were questions about self-rated health (or similar) with 3 or 5 response alternatives on an ordinal scale (19 studies) and the General Health Questionnaire (9 studies). A wide range of factors was included in the analyses of the relationship between health and unemployment. Gender and age were part of all but a few articles, and the most frequent other factors were education level (73%) and marital status (56%). Binary logistic regression was used in a majority of the studies (51%), and other regression techniques were also common (41%). Analysis of variance (ANOVA), analysis of covariance (ANCOVA), propensity scores, and instrumental variables were rarely found in our collection of articles.

**Table 1:** **Summary of characteristics for review articles**

Country	Year	Study design	Ages	#	Health measure	OR^a^	Gender	Age	Educ	MarS	Other factors	Method
Australia [17]	2001	CS	All^b^	7,682	Part of SF-36	n/a	Sep	Part	Part, Sep	Part	Ch, Geo, HHI, Other	LinReg (non-trivial)
Australia [26]	2001–2009	Long	20–55	7,176	MHI-5	n/a	Sep	Part	-	Part	Other	Path
Australia [32]	2001–2009	Long	15–64	37,369	MHI-5	n/a	Sep	Part	Part, Sep	Part	CM, HHI, SN, Other	LinReg (non-trivial)
Belgium [12]	1992–2002	Long	≤65	5,790	HDLFGDS	Age-based	Part	Sep	Part	Part^c^	HHI	Log
Brazil [16]	2002–2003	CS	15–64	6,426	SRH-5	1.7/1.3	Part	Part	Part	Part	Alc, HA, LC, Sm, Other	Log
Canada [9]	1994 & 1996	CS2	18–55	6,096	CIDI, DS	Age-based	Part	Sep	Part	Part	HA, Other	LinReg & Log
China [25]	2005	CS	20–49	8,075	HCTP	-/1.4	Part	Part, Sep	Part	Part	Alc, BMI, CM, Ethn, Geo, LC, PA, Sm, SN	Log (non-trivial)
Croatia [38]	1997–1999 & 2003	CS	19–57	4,139	SF-36	n/a	Part	Part	Part	-	-	ANCOVA
Europe [2]	2004	CS	50–64	11,462	SRH-5	-/2.1	Part	Part	Part	Part	Alc, BMI, Geo, PA, Sm, Other	Log
Europe [6]	2002–2004	CS	25–60	37,499	SRH-5	Strata	Sep	Part	-	-	Geo	Log
Europe [13]	1994–2002	Long	20–65	39,042 PY	Other	-/1.3	Part, Sep	Sep	Part	-	Geo, HHI	Log (non-trivial)
Europe [33]	1994/1995	CS	25–49	4,650	SRH-5	Strata^d^	Women	Part	-	Part	Ch, Geo, HHI	Log
Europe [34]	1994–1995	CS	25–49	6,449	SRH-3, SRH-5	Strata	Sep	Part	Part	Part	Ch, Geo	Log
Europe [36]	2008/2009	CS	First work < 30	5,746	EURO-D, SRH-5	Strata	Sep	Part	Part	-	Ethn, HA,LC, RU, SES, Other	Log
Finland [7]	1996–2001	Long	All^b^	20,599	SRH-5	n/a	Part	Part	Part	-	-	DiD, Prop
Finland [41]	1998	CS	20–54	15,468	BDI, SRH-5	Sex-based	Sep	Part	Part	Part	Alc, BMI, Sm, SN, Other	Log
Germany [23]	2009	CS	30–59	10,387	HRQOL-4	n/a	Sep	Part	Part	-	HHI, SN	MCDR
Germany [35]	1991–2008	Long	≤58	23,734	HS, MCSS	n/a	Part, Sep	Part, Sep	Part	Part	Ch, Geo, RU, SES, Other	LinReg (non-trivial
Great Britain [8]	1991–2008	Long	≥16	1,642	GHQ	n/a	Part	Part	Part	Part	HHI, LocUn, Other	LinReg (non-trivial)
Great Britain [10]	2003	CS	16–64	1,281	GHQ, SRH-5	1.5/1.7	Part	Part	Part	-	Geo	Log
Great Britain [14]	1991–2007	Long	16–65	10,494	GHQ	n/a	-	Part	Part	Part	HA, HHI, LC, Other	Reg
Great Britain [15]	1991–2009	Long	16–64	107,035	GHQ, Other	n/a	Part, Sep	Part, Sep	Part	Part	Ethn, HHI, LC, LocUn, Sm, Other	IV, LinReg
Great Britain [28]	2001	CS	25–59	698,880	SRH-3	n/a	Sep	Part	Part	-	LC	GLM
Great Britain [29]	1978–2004	CS2	25–59	264,660	SRH-3	Sex-based	Sep	Part	Part	-	LC, Other	Log
Great Britain [39]	1991–2009	Long	16–64	8,784	GHQ	n/a	Men	Part	-	Part	Ch, SES, Other	Reg
Norway [27]	1997–2002	Long	18–66	3,663	SCL	n/a	Part	Part	Part	Part	LocUn	Reg
Poland [22]	Not presented	CS^e^	25–64	968	EQ-VAS	2.7/1.5	Part, Sep	Part	Part	-	HHI, PA, Sm	Log
Slovakia [4]	1998 & 2002	CS2	~19–22	844	RAND, SRH-5, Well-being	n/a	Sep	-	-	-	CM, SN	Reg
Spain [3]	1994	CS	25–64	3,881	GHQ	Sex-based	Sep	Part	-	Sep	Ch, SES, Other	Log
Spain [30]	2006	CS	25–64	8,515	GHQ	n/a	Sep	Sep	-	-	SES, Other	Prev
Sweden [1]	1983–1989, 1992–1997	CS	16–64	59,571	SRH-5	1.8 & 2.4 /1.9 & 2.7^f^	Part, Sep	Part, Sep	Part, Sep	Part, Sep	Ethn, Geo, HA	MNLog
Sweden [5]	2001–2007	Long	20–59	12,605	GHQ	Strata	Part, Sep	Part, Sep	-	Part	Ch, HA, HHI, SES	Log
Sweden [18]	2007	Long	42	916	DepS, DS, SRH-3	Sex-based	Sep	Same age	-	-	HA	Log
Sweden [19]	1997	CS	18–24	3,453	Other	n/a	Sep^g^	Sep^g^	Sep^g^	-	CM, SN	ANOVA, t-test
Sweden [20]	1997	CS	25–64	4,149	Other	n/a	Sep^g^	Sep^g^	Sep^g^	-	CM, SN	ANOVA, t-test
Sweden [21]	1995	Long	30	1,044	DepS, SRH-3	Sex-based	Sep	Same age	-	-	CM, SN, Other	Log
Sweden [24]	1999–2000	CS	18–64	5,180	GHQ	Strata	Sep	Part	Part	-	CM, Geo, SN	Log
Sweden [31]	1995	Long	30	864	SRH-3	Sex-based	Sep	Same age	-	-	Alc, Ch, CM, HA, SES, SN, Other	Log
The Netherlands [37]	2003	CS	16–65	2,057	SF-36, SRH-5	-/2.6^d^	Part	Part	Part	Part	Ethn	Log, LinReg
USA [11]	Many	Long	Varies^h^	9,108	CES-D, SRH-5	n/a	Part	Part^i^	Part	Part	HHI, RU	LinReg
USA [40]	1999, 2001, 2003	Long	Unspecified	8,125; 16,724 PY	SRH-5	n/a	Part	Part	Part	Part	Ethn, HHI, RU, SES, Other	MNLog

*Explanation of short forms:*

**Country** refers to country where study was performed (Europe refers to studies where at least two European countries participated). **Year** refers to the year(s) the study was performed. **Study design** refers to the study design (CS = cross-sectional, CS2 = two cross sections of the same individuals, Long = longitudinal).

**Ages** = Age (in years) for participants; **#** = number of individuals in study population (PY = Person years).

**Health measures** BDI = 21-item version of Beck’s Depression Inventory (validated scale), CES-D = Centre for Epidemiological Studies Depression Scale (validated scale), CIDI = Composite International Diagnostic Interview, DepS = depressive symptoms, DS = distress scale, EURO-D = European collaboration (validated scale for depression), EQ-VAS = EuroQol 5-dimensions visual analogue scale (validated scale),GHQ = General Health Questionnaire (validated scale), HCTP = health compared with peers of same age, HDLFGDS = Health Daily Living Form Global Depression Scale (validated scale), HRQOL-4 = Four-item Health Days Core Module from the Center for Disease Control (validated scale), HS = health satisfaction, Index = author-created index, MCSS = Mental Component Summary Scale (part of SF-36), MHI = Mental Health Index (part of SF-36), RAND = SF-36 questions with a different scoring algorithm, SCL = Hopkins’ Symptom Check List (validated scale), SF-36 = Short Form 36 (validated scale), SRH = self-rated health (3 or 5 groups/alternatives in questionnaire))

**OR** = Odds ratio presented in the paper. The first number is the crude OR and the second is the OR from the multivariate model with the most variables if non-stratified estimates are presented (n/a = not applicable, − = odds ratio not estimated for uncontrolled multivariate model) **MarS** = Marital status used in analysis; **Sex** = Sex involved in the analysis; **Age** = Age used in the analysis.

**Other factors** = Other factors included as part of the analyses or in separate analyses (Alc = high alcohol consumption, BMI = body mass index, Ch = children in the household, CM = cash margin/financial stress, Ethn = ethnicity or other similar difference in personal characteristics, Geo = geographical comparisons within and between countries, HA = health aspects such as any chronic medical condition or long-standing illness, HHI = household or individual income, LC = living conditions, LocUn = local or regional unemployment, PA = physical activity, RU = reason for unemployment, SES = socioeconomic status based on work, Sm = smoking, SN = social networks/social).

**Method** = Statistical method used for analysing the relation between health and unemployment (ANOVA = analysis of variance, DiD = difference in difference (similar to linear regression), GLM = generalized linear models, IV = instrumental variables, Log = logistic regression, LinReg = linear regression, MCDR = multivariate count data regression, MNLog = multinomial logistic regression, OL = ordinary logit, Prop = propensity scores, Reg = regression technique other than linear and logistic).

For all parts of the matrix: Sep = separate analyses, Part = only included in the statistical model (odds ratio or similar not always presented).

^a^For all presented odds ratios in this column, health were significantly poorer for unemployed than employed individuals.

^b^Information about age is not explicitly stated.

^c^Variable “family composition” is not explained in the paper. We assume that the authors refer to marital status.

^d^Unemployment is added as a controlling factor, but in theory the odds ratio is calculated identically as if unemployment was the main exposure.

^e^Paper states that it has longitudinal variables but does not describe which year(s) the data collection is based on and the analysis does not indicate that it is based on longitudinal data.

^f^Odds ratios presented for the periods 1983–1989 and 1992–1997 in two separate analyses.

^g^Compares within groups of unemployed and employed but gives no direct comparison measurement and no significances for between-group comparisons.

^h^Two different populations are used in the article.

^i^Age was only available for one of the studies because the other had only individuals of the same age. No factors are included in both studies other than household income.

**Table 2:** **Characteristics of the articles in the review**

Characteristic (n = 41 articles)	n	%				
*Continent of study*						
Asia (China)	1	2.4				
Australia	3	7.3				
Europe^a^	33	80				
North America^b^	3	7.3				
South America (Brazil)	1	2.4				
*Study design*						
Cross-sectional	24	59				
Longitudinal	17	41				
*Health measure* ^*c*^						
Self-rated health	19	46				
General Health Questionnaire	9	22				
Depression scales	7	17				
Other health scales^d^	20	49				
			**Stratified analysis**		**Part of analysis**	
*Factors*			*n*	*%*	*n*	*%*
Gender^e^	38	93	25	66	19	50
Age^f^	37	90	11	30	31	84
Education level	30	73	5	17	28	93
Marital status	23	56	2	8.7	22	96
Household income	13	32	2	17	12	92
Geographic location	11	27	8	73	7	64
Social network/social support	10	24	4	40	8	80
Children at home	8	20	2	25	7	87
Cash margin/financial strain	8	20	2	25	6	75
Health aspects^g^	8	20	1	12	8	100
Socio-economic status	8	20	4	50	5	62
Living conditions and poverty	7	17	1	14	7	100
Ethnicity	6	15	2	33	6	100
Smoking	6	15	-	0	6	100
High alcoholic intake	5	12	1	0	5	100
Reason for unemployment	4	10	4	100	-	0
Local/regional unemployment rates	3	7.3	1	33	3	100
Overweight	3	7.3	-	0	3	100
Physical activity	3	7.3	-	0	3	100
*Statistical method* ^*h*^						
Binary logistic regression	21	51				
Other regression techniques	18	44				
ANOVA/ANCOVA	3	7.3				
Propensity scores	1	2.4				
Instrumental variables	1	2.4				
Prevalence ratios	1	2.4				

^a^Belgium (n = 1), Croatia (n = 1), Finland (n = 2), Germany (n = 2), Great Britain (n = 7), Norway (n = 1), Poland (n = 1), Slovakia (n = 1), Spain (n = 2), Sweden (n = 8), The Netherlands (n = 1), and collaborative studies between two or more European countries (n = 6).

^b^Canada (n = 1) and USA (n = 2).

^c^Ten studies included two health measurements and two studies included three health measurements.

^d^Three studies included two health measurements categorized as “other health scales” ^e^ One study only with men, one with only women, and one for which the statistical analysis method did not allow for using gender as a variable.

^f^Three studies included individuals of the same age and the fourth had similar ages ^g^Including previous health as well as current health-related issues such as any chronic medical condition or long-standing illness.

^h^Four studies presented results for two of the categories

Estimates of the health effect from unemployment for the general population

Almost half of the articles (n = 17) presented non-stratified results for the relationship between unemployment and health. Four articles presented an unadjusted odds ratio [1, 10, 16, 22], and the negative health effect ranged from 1.5 to 2.7 (Table 1). Odds ratios adjusted for one or more factors were presented in eight articles [1, 2, 10, 13, 16, 22, 25, 37] and ranged from 1.3 to 2.6. Thus, in all cases both the unadjusted and adjusted odds ratios showed significantly worse health for the unemployed. The four articles with unadjusted odds ratios also provided adjusted odds ratios. In two of the articles [16, 22] the odds ratios were lower for the adjusted and in the other two articles [1, 10] the adjusted odds ratios were higher compared with the unadjusted odds ratio.

Nine articles presented non-stratified results for the absolute difference between the groups of employed and unemployed individuals, and eight of these articles showed significantly poorer health for the unemployed [11, 19, 22, 37, 38, 40, 41, 45, 46]. The ninth article showed no indication of differences between the employed and unemployed and concluded that the differences shown in other studies are due to health selection [7]. The remaining 24 articles only presented stratified comparisons between the groups and/or no comparisons between employed and the heterogeneous group of unemployed (presenting results for two or more groups based on reason for unemployment).

### Gender

Three studies did not include gender in their analysis. One focused only on men [39], one focused only on women [33], and one used a fixed effects model for its statistical analysis that did not allow for time-invariant variables such as gender [14]. A majority of the 38 studies that included gender in their statistical analysis (66%) presented results of the effect of unemployment on health for men and women (Table 2). Among these studies, it was more common to find a more negative health effect due to unemployment for men [3, 5, 6, 13, 22–24, 28–30, 32] than for women [1, 15, 18, 31, 35, 36], and some studies found too small of a difference to reach a conclusion [17, 21, 26, 34, 41]. Comparisons between men and women were not reasonable for three of the studies [4, 19, 20].

In women, a significantly poorer health for the unemployed compared to the employed were commonly reported with adjusted odds ratios in the range from 1.5 to 2.5, while results among men varied more. The three studies that included countries from Eastern Europe showed a larger negative health effect for men than women from unemployment [4, 6, 22], and this was also the case for the two Spanish studies in our review that both showed a much larger numerical health effect from unemployment for men [3, 30]. However, it is notable that even though it was more common to see a more negative health effect in men than in women, the results for Swedish individuals more often showed that unemployed women [1, 18, 31, 34] were worse off than men [5, 24].

There were only a few cases where there was no significant negative effect from unemployment on health for either of the sexes. For women, such results were reported in Finland [34], Australia [32], and Spain [30]. For men, such results were reported in Sweden (at 30 years of age [31] and at 42 years of age [18]), in a multi-country European study looking at unemployment due to plant closures and layoffs [36], and in Denmark [34].

Thus, most studies have reported poorer health from unemployment in both men and women. How unemployment affects men and women varies between studies, but the majority of studies suggest that this is a bigger problem for men than for women.

### Age

Age was included in the analytic model or was presented with results on factor levels for most articles (37 of 41 studies). Four studies did not take age into consideration because all individuals in the studies were of similar age [18, 20, 21, 31]. Only a minority (n = 11) of the articles presented stratified results for the unemployment effect in different age groups (Table 2). In these studies, the age group that suffered the most from unemployment varied (Table 3) and depended on factors such as the reason for unemployment [35], country, and time period of the measurements. For example, there is less of a negative health effect in young Swedish unemployed adults in the more recent time period (1992–1997) than in the more past time period (1983–1989) [1].

**Table 3:** **The effect on health from unemployment within different age groups**

Country	Health measure	Results for age analysis in paper^a^		
Belgium [12]	Health Daily Living Form Global Depression Scale	20–29	OR: 3.2*	
		30–39	OR: 4.5*	
		40–49	OR: 2.4*	
		50–65	OR: 0.8 NS	
Canada [9]	Distress Scale	18–30	Difference: −0.05 NS	
		31–55	Difference: +0.20*	
China [25]	Health Compared To Peers	20–29	OR: 1.4*	
		30–39	OR: 1.2 NS	
		40–49	OR: 1.6*	
Spain [30]	General Health Questionnaire	25–34	Males	PR: 1.9 NS
			Females	PR: 1.4 NS
		35–44	Males	PR: 2.7*
			Females	PR: 0.6 NS
		45–54	Males	PR: 3.6*
			Females	PR: 1.5 NS
		55–64	Males	PR: 2.4*
			Females	PR: 1.5 NS
Great Britain [15]	General Health Questionnaire	16–29	Difference: +0.6^b^	
		30–39	Difference: +0.87^b^	
		40–49	Difference: +0.82^b^	
		50–64	Reference	
Germany [35]	Mental Component Summary Scale	50–58	Unemployed due to plant closure	Difference^c^: +1.629^d^
			Unemployed due to other reasons	Difference^c^: −0.21^d^
		All	Unemployed due to plant closure	Difference^c^: +0.492
			Unemployed due to other reasons	Difference^c^: −0.11
Sweden [1]	Self-rated health, 5 levels	16–25	Years: 1983–1989	OR: 3.8*
			Years: 1992–1997	OR: 2.6*
		26–45	Years: 1983–1989	OR: 1.6 NS
			Years: 1992–1997	OR: 3.4*
		46–64	Years: 1983–1989	OR: 1.3 NS
			Years: 1992–1997	OR: 2.8*
Sweden [5]	General Health Questionnaire	20–39	1–130 days of unemployment	OR: 1.1 NS
			More than 130 days of unemployment	OR: 1.2*
		40–59	1–130 days of unemployment	OR: 1.2 NS
			More than 130 days of unemployment	OR: 1.6*
Sweden [20]	Quality of life instrument	Comparisons are only made between age groups for unemployed and employed separately. Differences between age groups show no obvious age-related pattern.		
Sweden [19]	Quality of life instrument	Similar analysis performed as for study above		
European collaboration [13]	”Do you have any chronic physical or mental health problem, illness or disability?”	20–45	Odds ratio 1.31*	
		46–65	Odds ratio: 1.26*	

*Significant difference in health between unemployed and employed individuals in age group at the 5% level.

OR = odds ratio, PR = prevalence ratio, NS = no significant difference in health between unemployed and employed individuals in age group at 5% level.

^a^For all odds ratios and prevalence ratios, a ratio above 1 indicates a worse health effect for unemployed than employed individuals. The model with the most variables in it is chosen for all presentations in the table.

^b^Comparisons are not presented for health differences between employed and unemployed individuals.

^c^A positive difference means that the unemployed person tends to have better health.

^d^Results for age are presented as part of the tests for robustness of the analytical model.

### Socioeconomic factors

A clear majority of articles (73%) included education level in their analytical model (Table 2), but only three articles presented stratified results that allowed for comparisons on a factor level [1, 17, 32]. One of these studies was performed in Sweden [1], and this study found that for the years 1983 to 1989 there was significantly worse health for the unemployed than the employed among those having only primary or secondary education. In more recent years (1992–1997), significantly greater health effects were seen among those who were unemployed and who had secondary or post-secondary education. Inconclusive results were also seen in the two Australian studies that stratified for education level [17, 32].

Of 13 studies that involved household income, only two presented stratified results for wage groups. An Australian study showed that the negative effect due to employment on mental health was larger for men in the higher half of the salary range, but the opposite situation was seen for life satisfaction (which was not included as a health measure in our article) in both men and women [17]. In one Swedish study, it was only the group with the lowest salary among both the short- and long-term unemployed for whom unemployment led to significantly poorer health [5].

Socioeconomic status was included in eight articles and was most often defined as manual vs. non-manual workers. Half of the articles presented stratified results. In all articles, the pattern was that the manual workers had a greater negative health effect from unemployment than non-manual workers [3, 5, 30, 40], at least among the long-term unemployed [3, 5, 30].

Thus, socioeconomic status seems to be important in the health effect of unemployment, but it is not as clear what role education and salary have for the effect of unemployment on health.

### Family factors

Despite over half of the articles (56%) using marital status in their analyses, only two stratified the data based on this variable (Table 2). Both of these studies showed no apparent differences in health effect from unemployment between married persons and unmarried persons [1, 3]. A related topic is having a child at home. Not having a child at home was related to poorer health in both Swedish and Finnish women, but unemployment showed no difference in health effect between groups [33]. However, it was reported that Spanish women have significantly worse health if they have no child at home and have no unemployment benefits. Thus, having children might be a protective factor against unemployment-related health effects, at least under some circumstances [3].

### Social situation

Both a Chinese and a German study found tendencies that a strong social network was related to a smaller negative health effect due to unemployment [23, 25]. In the Chinese study, it was also shown that high alcohol consumption was related to a larger negative difference in health between unemployed and employed individuals [25]. However, the causality of the relation, i.e. whether the alcohol consumption was causing unemployment or unemployment was causing a higher alcohol intake, could not be proven due to the use of a cross-sectional study design.

### Geographical comparisons

In our review, there were five articles that presented results for two or more European countries [2, 6, 13, 33, 34] and three articles that presented results for different regions within the same country [1, 25, 35] (Table 2).

Cooper et al. presented odds ratios for the relationship between unemployment and health for ten European countries [13]. For four countries, the odds ratio was below 1.1 but for Greece and Austria it was above 2. Contrary to these results, it was shown in the study by Alavania et al. that there were no significant negative effects on health from unemployment in Greece and Austria [2], but there was a significant effect in Denmark even though Denmark had an odds ratio below 1 in the study by Cooper et al. There were also differences between countries in the other three multi-country studies [6, 33, 34].

The three studies that presented results from different areas within the same country showed that Sweden had a tendency for more negative health effects from unemployment in bigger cities [1], China had significantly worse health for the unemployed in two of three cities studied [25], and Germany had differences in effects between the eastern and western parts of the country [35].

Thus, the effect from unemployment on health varies between studies as well as within and between countries. Even estimates for the same countries can vary to a high extent between studies.

### Ethnicity and immigrant status

Two articles made comparisons based on the individual’s parent’s country of origin [1, 37]. A Swedish study compared health between unemployed and employed people born either in Sweden or somewhere else. There were negative health effects from unemployment for both time periods examined in that study for those born in Sweden, but negative health effects from unemployment were only seen in the latter time period (1993–1997) for those not born in Sweden (it should be noted that the odds ratio was still lower during this period than for those born in Sweden). Similar results were shown in the Netherlands where native Dutch and Antillean/Surinamese ethnic groups experienced a significant negative effect on health from unemployment, but not the Turkish/Moroccan and refugee groups (the size of the negative health effect from unemployment was as well larger in the first two groups) [37].

### Unemployment subgroups

Analyses based on reason for unemployment were stratified in four articles [11, 35, 36, 40] (Table 2). In an article based on US citizens that used two different study populations the unemployed were divided into two groups, one defined as being unemployed due to health reasons and the other being those who were unemployed for all other reasons [11]. In both of the populations, there was a more negative health effect among those who had lost their job due to health reasons in comparison with the employed. For those who lost their job due to other reasons than health issues, there was a small but significant negative health effect for the unemployed in one of the populations but not the other. In another US study, results were presented for white-collar and blue-collar workers. For blue-collar workers who were fired/laid-off or who had a voluntary job separation, there was a significantly greater health effect than for comparable white-collar workers, but the opposite was the case for those unemployed due to miscellaneous job separations [40]. Both in a German study and in a European multi-country study, the focus was mainly on the effect from job loss due to plant closure [35, 36]. The German study showed a small and non-significant positive health effect due to plant closure for both men and women, and the other study showed a negative effect from unemployment in all their groups, but the differences were not statistically significant for all groups.

Another interesting observation was made by Gathergood [15]. His model shows that the local unemployment rate in Great Britain affects the size of the health effect for the unemployed, with a lower unemployment rate being related to a larger negative health effect from unemployment.

## Discussion

Our review shows that unemployment affects people differently depending on the context in which a study has been performed. There were differences at the factor level for gender, age, education level, household income, and geographical location, but these varied between studies making firm conclusions difficult to support. Strong indicators of a more negative health effect due to unemployment were only seen for socioeconomic status (where the health of the manual workers was more negatively affected by unemployment), reason for unemployment (where the unemployed due to health reasons are worse off), and social network (where a strong social network was beneficial). However, only for age, gender, and geographic location were there more than eight studies that presented stratified results. It is therefore difficult to make firm conclusions on factor levels. Still, even if conclusions on factor levels can be drawn from the little evidence that exist, we can still conclude that the study context is of high importance for any results on how unemployment affects health.

Historically, the focus has been on describing whether people in general suffer a negative health effect from unemployment. We believe that it is of greater relevance to understand the extent to which unemployment causes health problems in different settings and for different factors instead of focusing on the effect size at the population level as was the case in the meta-analyses by McKee-Ryan et al. [45] and by Paul and Moser [46]. The balance of the factors we have investigated – as well as other factors such as the labour market system in a country – within the groups of unemployed and employed is a major issue in any analysis of the relation between unemployment and health. The health effect will, therefore, be the result of the composition of the population, and generalizations to larger communities – which the published meta-analyses attempt to do – might be misleading.

In our review, it was more common for studies to show a more negative health effect from unemployment for men than for women [3, 5, 6, 13, 22–24, 28–30, 32]. This might, at least in some studies, be explained by expectations for women not to have to work resulting in the man being the main income source for the family and, therefore, more vulnerable to unemployment. However, in some countries, such as Sweden, it is more common for women to be an active part of the work force than in some other countries [18], e.g. Spain during the 1990s. Thus, even if the Spanish studies show only a small negative health effect for women, this effect might not be explained by unemployment not having a negative effect on them [3, 30].

Age was also a factor for which the effect from unemployment on health varied. Paul and Moser showed in their meta-analysis signs of a U-shaped association between age and health problems due to unemployment that favoured middle-aged persons [46]. They did not present a detailed overview of their included studies and it is, therefore, not possible to judge whether they had a clear pattern within studies or if it was only the estimates from the meta-analysis that showed this pattern. We summarized the age-related odds ratios that have been presented in studies included in our present review, and we cannot support the proposal of a U-shaped effect size from unemployment. Instead we believe that how unemployment affects people of different ages will depend on the study context and will, therefore, vary between populations.

The results are also inconclusive for education level and salary. However, manual workers seem to suffer more from unemployment than non-manual workers, which perhaps can be considered contradictory to the inconclusive results for education level and salary. There are also indications of more serious negative health effects from unemployment for individuals with a poor social network.

Differences between and within countries illustrate that the health-related problems from unemployment cannot be seen as an equal burden in all contexts. Interestingly, there are indications that unemployment is less of a burden for immigrants [1, 37]. This might, however, be explained by both a generally poorer health for employed immigrants and the fact that immigrants usually have the least desirable jobs. Any conclusion regarding the effect on health from unemployment for immigrants should be treated with caution until further studies can be performed and until explanatory factors have been better analysed.

### Methodological considerations

The labour market has changed over time and the relevance of “old” data is lower for today’s situation which are the one we aim to describe in our review. We therefore restricted ourselves to articles published from 2003 and later. The year of publication is a good indicator of how recently data was collected in our review. The cut-off of 2003 meant that we covered all studies which included data collected from 1996 and more recently. There were 15 studies in our Web of Science search that were published from 1996 to 2002, but none of them included data from 1996 or more recently and fulfilled all other criteria for being included in our review. In the early 1990s there were a recession in many countries and to focus on the time after this would for such reason be logical. Among our 41 articles we had “old” data; 10 articles only included data from 1999 or earlier, and 15 articles included data collected before and after 1999, while one study lacked information about the year(s) data was collected. Thus, restricting ourselves to studies with data from no earlier than 2001 would have resulted in only 15 studies in our review, and extending the publication year to 1996 would not have added articles with more recent data than from 1995. We therefore think that our restriction to articles from no earlier than 2003 is motivated despite a change in pattern over time identified in some studies in our review [1, 3, 30].

The goal with our review was to include all relevant articles. We are aware that our search term in Web of Science might not have been sufficient for finding all relevant articles. We decided to perform our search based on the title of the article. An alternative approach would have been to extend the search to the topic of the article. This would have produced a list of around 90,000 articles from 2003 to 2013. There are also approaches which focus on extending the number of articles through previous searches such as the measure-driven approach suggested by Roelfs et al. [49]. However, alternative approaches are time demanding. Our search in PubMed, using MeSH terms, gave us only one additional article for the time period 2003 to 2013, which indicates that our search may be sufficient to find articles which target the effect on health from unemployment. We did not include articles without our key words in the title as they are likely to mainly target the relationship between health and a variable different than employment status. Including such articles could cause publication bias, as employment status might be removed from the statistical model if there is at most a marginal effect on health. Still, such articles might have contributed with important information for our review. We may still have a publication bias from our selection of articles, but we were not able to assess it because methods that assess publication bias require an effect measurement that is comparable for a reasonably large sample size. In our review, the number of articles which used the same effect measurement for the same health estimate was small.

It is an unavoidable problem in an observational study that the characteristics of two groups that are to be compared cannot be guaranteed to be similar to the same extent as is in a random controlled study. To overcome this problem most articles in our review are “controlling” for other factors in the statistical method. However, even though this creates comparable groups and unbiased estimates, the estimates will be highly affected by the composition of the study population. This becomes very obvious from the results that we present at the factor level. Longitudinal studies can better control for factors that occur before unemployment than cross-sectional studies can. We considered only including longitudinal studies, but the interpretation from our analysis is that the results at most differ marginally between the included longitudinal and cross-sectional studies.

Interestingly, the estimates for the unadjusted (ranging from 1.5 to 2.7) and adjusted (ranging from 1.3 to 2.6) estimates of the odds ratios for the effect on health from unemployment were rather similar [1, 2, 10, 13, 16, 22, 25, 37]. In the few studies that presented both adjusted and unadjusted estimates, the adjusted odds ratio were lower in two studies [1, 10], and the unadjusted odds ratio lower in the other two studies [16, 22]. Due to these similarities in estimates, it can be questioned whether studies in our review are correctly controlling for other factors. In most of the studies, we could not identify any reasoning about the role of these factors in the statistical model. Hence, estimates of the adjusted odds ratios in the articles might be biased rather than the adjusted and unadjusted estimates of the odds ratios not differing much.

We restricted our analyses on factor levels to studies which had presented stratified estimates. It would have been valuable to include all articles which used a factor in our analysis and such an approach is also common in meta-analyses. However, studies in our review differ in which variables are included in the statistical model and the study results depend on the study context. It would therefore be very complicated to derive representative estimates for differences on factor levels. Such advanced analysis is beyond the scope of this article. However, we think that there is support for our main conclusion without it.

Primarily the cross-sectional studies, but also the longitudinal studies, included in our review had difficulties in proving causality in the relationship between unemployment and health. Controlling for both health-related issues and other factors before unemployment will improve estimates. The interpretations in our review have not put much emphasis on the causality problem, but we do think that the conclusions from our review are still valid.

The articles in the review used a variety of instruments to collect information about self-assessed health. Most common was self-rated health, which was used in almost half (n = 19) of the studies, and it was measured with both five (n = 14) and three (n = 6) response alternatives. Thus, for all health measurements it was at most a small number of studies using it. Despite the difficulties in comparing the measurements, we find a value of including any self-assessed measurement to provide all relevant evidence for the effect on health from unemployment (both with and without stratification), even if one health measurement would have been more frequently used. The wide range of health measures for self-assessed health is a weakness which might alter some of our interpretations in the result section. However, we are convinced that the conclusions of our review are not much affected by this limitation.

Morbidity-related measurements are usually a good indicator of how individuals rate their health. However, it is well-known that even an individual with a severe disease can consider their health to be good. Hence, morbidity-related measurements might not refer to how well the individual feels, and therefore differ too much from our definition of self-assessed health. Neither do we think that mortality is possible to compare with self-assessed health as it is a rare and more severe outcome. Actually, we propose that it would be valuable to perform a similar approach as ours to morbidity-related measurements and mortality in relation to unemployment, but this is beyond the scope of our review.

We extracted relevant information from the articles in our review (see Table 1). However, the quality of the methods sections of the articles varied and we cannot with certainty confirm that everything has been interpreted correctly. These problems included not only difficulties in interpreting the study design and the statistical methods, but also problems with the documentation of the variable specifications. For example, no specification of family composition was provided in one of the studies [12] and no mention of the included ages was made in another study [17]. This is a weakness in our review, but we believe that these shortcomings might have only a slight effect on our conclusions.

## Conclusions

We have shown that unemployment affects groups of individuals differently. Still, there are a few factors, among them socioeconomic status, reason for unemployment, and social network, where a clear pattern can be seen. For gender, age, and education level, which are most commonly stratified for, the results differ between studies and seem to depend to a large degree on the study context. Because unemployment affects groups differently, we believe that greater effort should be spent on groups of individuals, such as men or women, instead of the population as a whole.

## Electronic supplementary material

Additional file 1: **PRISMA 2009 Checklist.** (DOC 64 KB)

Additional file 2: **Selection of articles.** (DOCX 185 KB)
