# Supplementary material for: How does unemployment affect self-assessed health? A systematic review focusing on subgroup effects
Source: BMC Public Health. 2014 Dec 22;14:1310. doi: 10.1186/1471-2458-14-1310 (PMC4364585; doi:10.1186/1471-2458-14-1310)
Supplement: Supplementary file 2 — Additional file 2: Selection of articles. (DOCX 185 KB) [file 12889_2014_7438_MOESM2_ESM.docx]

# Appendix 1 – selection of articles

The identification of articles for our study was based mainly on a search in Web Of Science (Thomson Reuters). In our first identification of articles, the database was scanned on the 26^th^ of July 2013. The search term used for this scanning was ("well-being" OR "health") AND ("labor" or "labour" or "employment" or "job" or "unemployment" or "work" or "unemployed"), and the search only included articles whose titles matched the search term. The first part of the search term was related to the health definition and the second part to the employment status definition. The scanning, which was not limited to any particular parameter, e.g. publication year or language, resulted in 8,070 articles.

In the first step we removed articles based on their titles, resulting in only 412 articles with a title that at least to some extent indicated that the article had content that might match our research question. In the second step we required that the articles were written in English (n = 18 articles excluded) and were defined by Web of Science as document type “article” (n = 132 articles excluded, including 20 “book review”, 27 “editorial material”, 30 “letter”, 39 “meeting abstract” and 16 “proceedings paper”). For the remaining 262 articles, the abstracts were read and a further 160 articles were removed because the content showed that the article did not fulfil our inclusion criteria [[1-102](#_ENREF_1)]. We restricted ourselves to the 58 [[2-4](#_ENREF_2), [6-9](#_ENREF_6), [11](#_ENREF_11), [13-21](#_ENREF_13), [24](#_ENREF_24), [30](#_ENREF_30), [32-35](#_ENREF_32), [37](#_ENREF_37), [39](#_ENREF_39), [41](#_ENREF_41), [44](#_ENREF_44), [47](#_ENREF_47), [49](#_ENREF_49), [50](#_ENREF_50), [53](#_ENREF_53), [54](#_ENREF_54), [59](#_ENREF_59), [61](#_ENREF_61), [63](#_ENREF_63), [68-75](#_ENREF_68), [77](#_ENREF_77), [78](#_ENREF_78), [80](#_ENREF_80), [82-84](#_ENREF_82), [86](#_ENREF_86), [87](#_ENREF_87), [89](#_ENREF_89), [92-98](#_ENREF_92)] of the remaining 102 articles that were published from 2003 and onwards. After reading these articles in full, we kept the 36 articles [[2](#_ENREF_2), [3](#_ENREF_3), [6-9](#_ENREF_6), [13-16](#_ENREF_13), [18](#_ENREF_18), [19](#_ENREF_19), [21](#_ENREF_21), [32](#_ENREF_32), [33](#_ENREF_33), [37](#_ENREF_37), [44](#_ENREF_44), [49](#_ENREF_49), [50](#_ENREF_50), [54](#_ENREF_54), [59](#_ENREF_59), [61](#_ENREF_61), [68](#_ENREF_68), [70-72](#_ENREF_70), [74](#_ENREF_74), [75](#_ENREF_75), [77](#_ENREF_77), [78](#_ENREF_78), [82-84](#_ENREF_82), [86](#_ENREF_86), [89](#_ENREF_89), [94](#_ENREF_94)] that had a content in line with our research question, i.e. articles that measured how unemployment affects health.

We extended our search term for health to include “quality of life”, thus the updated search term was ("well-being" OR "health" OR “quality of life” ) AND ("labor" or "labour" or "employment" or "job" or "unemployment" or "work" or "unemployed" or “contract”), which resulted in one additional article [[103](#_ENREF_103)]. We followed-up with weekly emails from the database with the extended search term until the 6th of April 2014, and this resulted in three additional articles [[104-106](#_ENREF_104)]. To improve our chances to include all relevant articles, the literature database PubMed (National Center for Biotechnology Information, Bethesda MD, USA) was also scanned. We used the search terms “employ*” and “health” and these terms had to be part of the article’s MeSH terms. This search only resulted in one additional article [[107](#_ENREF_107)] that fulfilled our criteria and was not available in Web of Science. Thus, the final selection of 41 articles included 36 from the initial search of the Web Of Science database, one from an extended search term, three published after the initial search, and one that was only available on PubMed.

# References

1. Adelmann PK, Antonucci TC, Crohan SE, Coleman LM: **A Causal-Analysis of Employment and Health in Midlife Women**. *Women Health* 1990, **16**(1):5-20.

2. Åhs A, Westerling R: **Self-rated health in relation to employment status during periods of high and of low levels of unemployment**. *Eur J Public Health* 2006, **16**(3):294-304.

3. Alavinia SM, Burdorf A: **Unemployment and retirement and ill-health: a cross-sectional analysis across European countries**. *Int Arch Occ Env Hea* 2008, **82**(1):39-45.

4. Andersen SH: **Unemployment and Subjective Well-Being A Question of Class?** *Work Occupation* 2009, **36**(1):3-25.

5. Arrow JO: **Estimating the influence of health as a risk factor on unemployment: A survival analysis of employment durations for workers surveyed in the German Socio-Economic Panel (1984-1990)**. *Soc Sci Med* 1996, **42**(12):1651-1659.

6. Artazcoz L, Benach J, Borrell C, Cortes I: **Unemployment and mental health: Understanding the interactions among gender, family roles, and social class**. *Am J Public Health* 2004, **94**(1):82-88.

7. Bacikova-Sleskova M, van Dijk JP, Geckova AM, Nagyova I, Salonna F, Reijneveld SA, Groothoff JW: **The impact of unemployment on school leavers' perception of health. Mediating effect of financial situation and social contacts?** *Int J Public Health* 2007, **52**(3):180-187.

8. Backhans MC, Hemmingsson T: **Unemployment and mental health-who is (not) affected?** *Eur J Public Health* 2012, **22**(3):429-433.

9. Bambra C, Eikemo TA: **Welfare state regimes, unemployment and health: a comparative study of the relationship between unemployment and self-reported health in 23 European countries**. *J Epidemiol Commun H* 2009, **63**(2):92-98.

10. Bellaby P, Bellaby F: **Unemployment and ill health: Local labour markets and ill health in Britain 1984-1991**. *Work Employ Soc* 1999, **13**(3):461-482.

11. Bernhard-Oettel C, Sverke M, De Witte H: **Comparing three alternative types of employment with permanent full-time work: How do employment contract and perceived job conditions relate to health complaints?** *Work Stress* 2005, **19**(4):301-318.

12. Björklund A: **Unemployment and Mental-Health - Some Evidence from Panel Data**. *J Hum Resour* 1985, **20**(4):469-483.

13. Böckerman P, Ilmakunnas P: **Unemployment and Self-Assessed Health: Evidence from Panel Data**. *Health Econ* 2009, **18**(2):161-179.

14. Booker CL, Sacker A: **Psychological well-being and reactions to multiple unemployment events: adaptation or sensitisation?** *J Epidemiol Commun H* 2012, **66**(9):832-838.

15. Breslin FC, Mustard C: **Factors influencing the impact of unemployment on mental health among young and older adults in a longitudinal, population-based survey**. *Scand J Work Env Hea* 2003, **29**(1):5-14.

16. Brown J, Demou E, Tristram MA, Gilmour H, Sanati KA, Macdonald EB: **Employment status and health: understanding the health of the economically inactive population in Scotland**. *Bmc Public Health* 2012, **12**.

17. Burgard SA, Kalousova L, Seefeldt KS: **Perceived Job Insecurity and Health The Michigan Recession and Recovery Study**. *J Occup Environ Med* 2012, **54**(9):1101-1106.

18. Burgard SA, Brand JE, House JS: **Toward a better estimation of the effect of job loss on health**. *J Health Soc Behav* 2007, **48**(4):369-384.

19. Burnay N, Kiss P, Malchaire J: **Sociability, life satisfaction, and mental health according to age and (un)employment status**. *Int Congr Ser* 2005, **1280**:347-352.

20. Chatterji P, Markowitz S, Brooks-Gunn J: **Effects of early maternal employment on maternal health and well-being**. *J Popul Econ* 2013, **26**(1):285-301.

21. Cooper D, McCausland WD, Theodossiou I: **Unemployed, uneducated and sick: the effects of socio-economic status on health duration in the European Union**. *J R Stat Soc a Stat* 2008, **171**:939-952.

22. Darcy C, Siddique CM: **Unemployment and Health - an Analysis of Canada Health Survey Data**. *Int J Health Serv* 1985, **15**(4):609-635.

23. Darcy C: **Unemployment and Health - Data and Implications**. *Can J Public Health* 1986, **77**:124-131.

24. Dean JA, Wilson K: **'Education? It is irrelevant to my job now. It makes me very depressed ': exploring the health impacts of under/unemployment among highly skilled recent immigrants in Canada**. *Ethnic Health* 2009, **14**(2):185-204.

25. Dew MA, Penkower L, Bromet EJ: **Effects of Unemployment on Mental-Health in the Contemporary Family**. *Behav Modif* 1991, **15**(4):501-544.

26. Donovan A, Oddy M, Pardoe R, Ades A: **Employment Status and Psychological Well-Being - a Longitudinal-Study of 16-Year-Old School Leavers**. *J Child Psychol Psyc* 1986, **27**(1):65-76.

27. Dooley D, Fielding J, Levi L: **Health and unemployment**. *Annu Rev Publ Health* 1996, **17**:449-465.

28. Farrow SC: **Monitoring the Health-Effects of Unemployment**. *J Roy Coll Phys Lond* 1983, **17**(2):99-&.

29. Ferrie JE, Shipley MJ, Stansfeld SA, Marmot MG: **Effects of chronic job insecurity and change in job security on self reported health, minor psychiatric morbidity, physiological measures, and health related behaviours in British civil servants: the Whitehall II study**. *J Epidemiol Commun H* 2002, **56**(6):450-454.

30. Fritzell S, Vannoni F, Whitehead M, Burström B, Costa G, Clayton S, Fritzell J: **Does non-employment contribute to the health disadvantage among lone mothers in Britain, Italy and Sweden? Synergy effects and the meaning of family policy**. *Health Place* 2012, **18**(2):199-208.

31. Gallo WT, Bradley EH, Siegel M, Kasl SV: **Health effects of involuntary job loss among older workers: Findings from the health and retirement survey**. *J Gerontol B-Psychol* 2000, **55**(3):S131-S140.

32. Gathergood J: **An Instrumental Variable Approach to Unemployment, Psychological Health and Social Norm Effects**. *Health Econ* 2013, **22**(6):643-654.

33. Giatti L, Barreto SM, Cesar CC: **Unemployment and self-rated health: Neighborhood influence**. *Soc Sci Med* 2010, **71**(4):815-823.

34. Gordo LR: **Effects of short- and long-term unemployment on health satisfaction: evidence from German data**. *Appl Econ* 2006, **38**(20):2335-2350.

35. Gracia FJ, Ramos J, Peiro JM, Caballer A, Sora B: **Job attitudes, behaviours and well-being among different types of temporary workers in Europe and Israel**. *Int Labour Rev* 2011, **150**(3-4):235-254.

36. Graetz B: **Health Consequences of Employment and Unemployment - Longitudinal Evidence for Young Men and Women**. *Soc Sci Med* 1993, **36**(6):715-724.

37. Green F: **Unpacking the misery multiplier: How employability modifies the impacts of unemployment and job insecurity on life satisfaction and mental health**. *J Health Econ* 2011, **30**(2):265-276.

38. Hagquist C, Starrin B: **Youth unemployment and mental health - Gender differences and economic stress**. *Scand J Soc Welfare* 1996, **5**(4):215-228.

39. Haid ML, Seiffge-Krenke I: **Effects of (un)employment on young couples' health and life satisfaction**. *Psychol Health* 2013, **28**(3):284-301.

40. Hamilton VH, Merrigan P, Dufresne E: **Down and out: Estimating the relationship between mental health and unemployment**. *Health Econ* 1997, **6**(4):397-406.

41. Hammarström A, Virtanen P, Janlert U: **Are the health consequences of temporary employment worse among low educated than among high educated?** *Eur J Public Health* 2011, **21**(6):756-761.

42. Hammarström A, Janlert U: **Early unemployment can contribute to adult health problems: results from a longitudinal study of school leavers**. *J Epidemiol Commun H* 2002, **56**(8):624-630.

43. Hammarström A: **Health Consequences of Youth Unemployment**. *Public Health* 1994, **108**(6):403-412.

44. Hammarström A, Gustafsson PE, Strandh M, Virtanen P, Janlert U: **It's no surprise! Men are not hit more than women by the health consequences of unemployment in the Northern Swedish Cohort**. *Scand J Public Healt* 2011, **39**(2):187-193.

45. Hammarström A, Janlert U, Theorell T: **Youth Unemployment and Ill Health - Results from a 2-Year Follow-up-Study**. *Soc Sci Med* 1988, **26**(10):1025-1033.

46. Hammer T: **Unemployment and Mental-Health among Young-People - a Longitudinal-Study**. *J Adolescence* 1993, **16**(4):407-420.

47. Hintikka J, Lehto SM, Niskanen L, Huotari A, Herzig KH, Koivumaa-Honkanen H, Honkalampi K, Sinikallio S, Viinamäki H: **Unemployment and ill health: a connection through inflammation?** *Bmc Public Health* 2009, **9**.

48. Jackson PR, Warr PB: **Unemployment and Psychological Ill-Health - the Moderating Role of Duration and Age**. *Psychol Med* 1984, **14**(3):605-614.

49. Janlert U, Hammarström A: **Which theory is best? Explanatory models of the relationship between unemployment and health**. *Bmc Public Health* 2009, **9**.

50. Kaleta D, Makowiec-Dabrowska T, Jegier A: **Employment Status and Self Rated Health**. *Int J Occup Med Env* 2008, **21**(3):227-236.

51. Keith PM, Schafer RB: **Employment Status, Household Involvement, and Psychological Well-Being of Men and Women**. *Int J Sociol Fam* 1982, **12**(1):101-110.

52. Kessler RC, House JS, Turner JB: **Unemployment and Health in a Community Sample**. *J Health Soc Behav* 1987, **28**(1):51-59.

53. Kompier M, Ybema JF, Janssen J, Taris T: **Employment Contracts: Cross-sectional and Longitudinal Relations with Quality of Working Life, Health and Well-being**. *J Occup Health* 2009, **51**(3):193-203.

54. Kroll LE, Lampert T: **Unemployment, Social Support and Health Problems Results of the GEDA Study in Germany, 2009**. *Dtsch Arztebl Int* 2011, **108**(4):47-U14.

55. Kulenovic M, Abramovic Z: **Quality-of-Life and Mental Well-Being of the Unemployed**. *Collegium Antropol* 1995, **19**(1):171-177.

56. Lahelma E: **Unemployment and Mental Well-Being - Elaboration of the Relationship**. *Int J Health Serv* 1992, **22**(2):261-274.

57. Lai JCL, Chan RKH, Luk CL: **Unemployment and psychological health among Hong Kong Chinese women**. *Psychol Rep* 1997, **81**(2):499-505.

58. Layton C: **Employment, Unemployment, and Response to the General Health Questionnaire**. *Psychol Rep* 1986, **58**(3):807-810.

59. Lindström M: **Psychosocial work conditions, unemployment and self-reported psychological health: a population-based study**. *Occup Med-Oxford* 2005, **55**(7):568-571.

60. Linn MW, Sandifer R, Stein S: **Effects of Unemployment on Mental and Physical Health**. *Am J Public Health* 1985, **75**(5):502-506.

61. Luo J, Qu Z, Rockett I, Zhang X: **Employment status and self-rated health in north-western China**. *Public Health* 2010, **124**(3):174-179.

62. Mastekaasa A: **Unemployment and health: Selection effects**. *J Community Appl Soc* 1996, **6**(3):189-205.

63. McKee-Ryan FM, Song ZL, Wanberg CR, Kinicki AJ: **Psychological and physical well-being during unemployment: A meta-analytic study**. *J Appl Psychol* 2005, **90**(1):53-76.

64. Muller J, Hicks R, Winocur S: **The Effects of Employment and Unemployment on Psychological Well-Being in Australian Clerical Workers - Gender Differences**. *Aust J Psychol* 1993, **45**(2):103-108.

65. Murphy GC, Athanasou JA: **The effect of unemployment on mental health**. *J Occup Organ Psych* 1999, **72**:83-99.

66. Novo M, Hammarström A, Janlert U: **Do high levels of unemployment influence the health of those who are not unemployed? A gendered comparison of young men and women during boom and recession**. *Soc Sci Med* 2001, **53**(3):293-303.

67. Novo M, Hammarström A, Janlert U: **Health hazards of unemployment - only a boom phenomenon? A study of young men and women during times of prosperity and times of recession**. *Public Health* 2000, **114**(1):25-29.

68. Østhus S: **Health effects of downsizing survival and job loss in Norway**. *Soc Sci Med* 2012, **75**(5):946-953.

69. Paul KI, Moser K: **Unemployment impairs mental health: Meta-analyses**. *J Vocat Behav* 2009, **74**(3):264-282.

70. Popham F, Gray L, Bambra C: **Employment status and the prevalence of poor self-rated health. Findings from UK individual-level repeated cross-sectional data from 1978 to 2004**. *Bmj Open* 2012, **2**(6).

71. Popham F, Bambra C: **Evidence from the 2001 English Census on the contribution of employment status to the social gradient in self-rated health**. *J Epidemiol Commun H* 2010, **64**(3):277-280.

72. Puig-Barrachina V, Malmusi D, Martinez JM, Benach J: **Monitoring Social Determinants of Health Inequalities: The Impact of Unemployment among Vulnerable Groups**. *Int J Health Serv* 2011, **41**(3):459-482.

73. Reine I, Novo M, Hammarström A: **Does the association between ill health and unemployment differ between young people and adults? Results from a 14-year follow-up study with a focus on psychological health and smoking**. *Public Health* 2004, **118**(5):337-345.

74. Reine I, Novo M, Hammarström A: **Unemployment and ill health - A gender analysis: Results from a 14-year follow-up of the Northern Swedish Cohort**. *Public Health* 2013, **127**(3):214-222.

75. Richardson S, Lester L, Zhang GY: **Are Casual and Contract Terms of Employment Hazardous for Mental Health in Australia?** *J Ind Relat* 2012, **54**(5):557-578.

76. Rodriguez E: **Marginal employment and health in Britain and Germany: does unstable employment predict health?** *Soc Sci Med* 2002, **55**(6):963-979.

77. Roos E, Lahelma E, Saastamoinen P, Elstad JI: **The association of employment status and family status with health among women and men in four Nordic countries**. *Scand J Public Healt* 2005, **33**(4):250-260.

78. Roos E, Burström B, Saastamoinen P, Lahelma E: **A comparative study of the patterning of women's health by family status and employment status in Finland and Sweden**. *Soc Sci Med* 2005, **60**(11):2443-2451.

79. Ross CE, Mirowsky J: **Does Employment Affect Health**. *J Health Soc Behav* 1995, **36**(3):230-243.

80. Samuelsson A, Houkes I, Verdonk P, Hammarström A: **Types of employment and their associations with work characteristics and health in Swedish women and men**. *Scand J Public Healt* 2012, **40**(2):183-190.

81. Schaufeli WB: **Youth unemployment and mental health: Some Dutch findings**. *J Adolescence* 1997, **20**(3):281-292.

82. Schmitz H: **Why are the unemployed in worse health? The causal effect of unemployment on health**. *Labour Econ* 2011, **18**(1):71-78.

83. Schröder M: **Jobless now, sick later? Investigating the long-term consequences of involuntary job loss on health**. *Adv Life Course Res* 2013, **18**(1):5-15.

84. Schuring M, Burdorf A, Kunst A, Voorham T, Mackenbach J: **Ethnic differences in unemployment and ill health**. *Int Arch Occ Env Hea* 2009, **82**(8):1023-1030.

85. Schwefel D, John J, Potthoff P, Hechler A: **Unemployment and Mental-Health - Perspectives from the Federal-Republic-of-Germany**. *Int J Ment Health* 1984, **13**(1-2):35-50.

86. Sersic DM, Sverko B, Galesic M: **Unemployment and dimensions of subjective health: Exploring moderating effects of age**. *Stud Psychol* 2005, **47**(3):221-234.

87. Silla I, Gracia FJ, Peiro JM: **Job insecurity and health-related outcomes among different types of temporary workers**. *Econ Ind Democracy* 2005, **26**(1):89-117.

88. Söderstrom L: **Some Effects of Unemployment on the Health of Unemployed Quebec Workers**. *Relat Ind-Ind Relat* 1988, **43**(2):341-377.

89. Strully KW: **Job Loss and Health in the Us Labor Market**. *Demography* 2009, **46**(2):221-246.

90. Viinamäki H, Koskela K, Niskanen L: **Rapidly declining mental well-being during unemployment**. *Eur J Psychiat* 1996, **10**(4):215-221.

91. Virtanen P, Vahtera J, Kivimäki M, Pentti J, Ferrie J: **Employment security and health**. *J Epidemiol Commun H* 2002, **56**(8):569-574.

92. Virtanen P, Janlert U, Hammarström A: **Exposure to Nonpermanent Employment and Health Analysis of the Associations With 12 Health Indicators**. *J Occup Environ Med* 2011, **53**(6):653-657.

93. Virtanen P, Janlert U, Hammarström A: **Exposure to temporary employment and job insecurity: a longitudinal study of the health effects**. *Occup Environ Med* 2011, **68**(8):570-574.

94. Virtanen P, Liukkonen V, Vahtera J, Kivimäki M, Koskenvuo M: **Health inequalities in the workforce: the labour market core-periphery structure**. *Int J Epidemiol* 2003, **32**(6):1015-1021.

95. Virtanen P, Saloniemi A, Vahtera J, Kivimäki M, Virtanen M, Koskenvuo M: **The working conditions and health of non-permanent employees: Are there differences between private and public labour markets?** *Econ Ind Democracy* 2006, **27**(1):39-65.

96. Waenerlund AK, Virtanen P, Hammarström A: **Is temporary employment related to health status? Analysis of the Northern Swedish Cohort**. *Scand J Public Healt* 2011, **39**(5):533-539.

97. Waenerlund AK, Gustafsson PE, Virtanen P, Hammarström A: **Is the core-periphery labour market structure related to perceived health? findings of the Northern Swedish Cohort**. *Bmc Public Health* 2011, **11**.

98. Wagenaar AF, Kompier MAJ, Houtman ILD, van den Bossche S, Smulders P, Taris TW: **Can labour contract differences in health and work-related attitudes be explained by quality of working life and job insecurity?** *Int Arch Occ Env Hea* 2012, **85**(7):763-773.

99. Whiteside N: **Unemployment and Health - an Historical-Perspective**. *J Soc Policy* 1988, **17**:177-194.

100. Winefield AH: **Employment History and Psychological Well-Being in the Young Unemployed**. *Psychol Rep* 1993, **72**(1):14-14.

101. Winefield AH, Tiggemann M: **Employment Status and Psychological Well-Being - a Longitudinal-Study**. *J Appl Psychol* 1990, **75**(4):455-459.

102. Winefield AH, Tiggemann M: **Unemployment Duration and Affective Well-Being in the Young**. *J Occup Psychol* 1989, **62**(4):327-336.

103. Hultman B, Hemlin S: **Self-rated quality of life among the young unemployed and the young in work in northern Sweden**. *Work* 2008, **30**(4):461-472.

104. Flint E, Bartley M, Shelton N, Sacker A: **Do labour market status transitions predict changes in psychological well-being?** *J Epidemiol Community Health* 2013, **67**(9):796-802.

105. Olesen SC, Butterworth P, Leach LS, Kelaher M, Pirkis J: **Mental health affects future employment as job loss affects mental health: findings from a longitudinal population study**. *Bmc Psychiatry* 2013, **13**.

106. Steele F, French R, Bartley M: **Adjusting for Selection Bias in Longitudinal Analyses Using Simultaneous Equations Modeling The Relationship Between Employment Transitions and Mental Health**. *Epidemiology* 2013, **24**(5):703-711.

107. Hultman B, Hemlin S, Hörnquist JO: **Quality of life among unemployed and employed people in northern Sweden. Are there any differences?** *Work (Reading, Mass)* 2006, **26**(1):47-56.
